# Ultra-processed food consumption and dental pain in adolescents: a descriptive analysis, Brazil, 2019

**DOI:** 10.1590/S2237-96222026v35e20250508.en

**Published:** 2026-07-06

**Authors:** Nathalia Ribeiro Jorge da Silva-Garcia, Sarah Arangurem Karam, Eduardo Dickie de Castilhos, Mariana Gonzalez Cademartori

**Affiliations:** 1 Universidade Federal de Pelotas Programa de Pós-Graduação em Odontologia Pelotas RS Brazil Universidade Federal de Pelotas, Programa de Pós-Graduação em Odontologia, Pelotas, RS, Brazil; 2 Universidade Católica de Pelotas Programa de Pós-Graduação em Saúde no Ciclo Vital Pelotas RS Brazil Universidade Católica de Pelotas, Programa de Pós-Graduação em Saúde no Ciclo Vital, Pelotas, RS, Brazil

**Keywords:** Dental Pain, Eating, Adolescents, Food, Ultra-processed, Public Health Surveillance, Dolor Dental, Ingestión de Alimentos, Adolescentes, Alimentos Ultraprocesados, Vigilancia en Salud Pública

## Abstract

**Objective:**

To analyze association between ultra-processed food consumption and self-reported dental pain by Brazilian adolescents.

**Methods:**

This is a cross-sectional study with data from the 2019 National School Health Survey with adolescents aged 13 to 17 years. The outcome was self-reported dental pain in the last six months. Ultra-processed food consumption was assessed based on the frequency of intake in the last 24 hours and in the last seven days, according to the NOVA classification, considering consumption of sweet food, soft drinks and fast-food meals categorized as low (0-2 days), moderate (3-4 days) or high (5-7 days). Associations were estimated using prevalence ratios (PR) adjusted for sex, age, race/skin color, maternal education and dentist visit.

**Results:**

Of the 124,898 adolescents analyzed, 21.5% reported dental pain. 98.4% consumed ultra-processed food in the last 24 hours. Low consumption of sweet food (40.5%), soda (61.9%) and fast-food meals (82.1%) was observed in terms of weekly frequency. In the adjusted analysis, dental pain prevalence was higher among adolescents with high consumption of sweet food (PR 1.27; 95%CI 1.22; 1.31), soda (PR 1.28; 95%CI 1.23; 1.32) and fast-food meals (PR 1.22; 95%CI 1.15; 1.29) in the last week, compared to those with low consumption.

**Conclusion:**

Higher frequency of ultra-processed food consumption is associated with higher prevalence of dental pain among Brazilian adolescents, highlighting the relevance of intersectoral strategies for promoting healthy eating and oral health prevention in this age group.

## Resumen

**Objetivo:**

Analizar la asociación entre el consumo de alimentos ultraprocesados ​​y el reporte de dolor dental en adolescentes brasileños.

**Métodos:**

Estudio transversal con datos de la Encuesta Nacional de Salud Escolar de 2019, con adolescentes de 13 a 17 años. El resultado fue el autoreporte de dolor dental en los últimos seis meses. El consumo de alimentos ultraprocesados ​​se evaluó con base en la frecuencia de ingesta en las últimas 24 horas y en los últimos siete días, según la clasificación NOVA, considerando el consumo de dulces, refrescos y comidas rápidas categorizado como bajo (0-2 días), moderado (3-4 días) o alto (5-7 días). Las asociaciones se estimaron mediante razones de prevalencia (RP) ajustadas por sexo, edad, raza/color de piel, educación materna y consulta al dentista.

**Resultados:**

De los 124,898 adolescentes analizados, el 21.5% reportó dolor dental. El 98.4% consumió alimentos ultraprocesados ​​en las últimas 24 horas. Se observó un bajo consumo de dulces (40,5%), refrescos (61,9%) y comida rápida (82,1%) en términos de frecuencia semanal. En el análisis ajustado, la prevalencia de dolor dental fue mayor entre los adolescentes con un alto consumo de dulces (RP 1,27; IC95% 1,22; 1,31), refrescos (RP 1,28; IC95% 1,23; 1,32) y comida rápida (RP 1,22; IC95% 1,15; 1,29) en la última semana, en comparación con aquellos con un bajo consumo.

**Conclusión:**

Un mayor consumo de alimentos ultraprocesados ​​se asocia con una mayor prevalencia de dolor dental entre los adolescentes brasileños, lo que refuerza la importancia de las estrategias intersectoriales para promover una alimentación saludable y la prevención de la salud bucodental en este grupo de edad.

**Ethical aspects**

This research used public domain anonymized databases.

## Introduction

The global increase in ultra-processed food consumption has been shown to be a significant risk factor for chronic non-communicable diseases [1]. Recognizing that inadequate dietary habits represent a significant public health concern and adversely affect various health outcomes, the World Health Organization has recommended reducing consumption of these products, especially in the case of children and adolescents, these being critical periods for the establishment of long-term dietary patterns [2,3]. Research focused on these developmental stages is therefore of paramount importance [4,5].

Brazilian studies show high prevalence of dental pain in adolescents: 19% of 12-year-old adolescents in the state of Minas Gerais [8] and 30% of adolescents aged 15 to 19 in the southern region of Brazil [9]. Another study, in the context of the Family Health Strategy in Manaus city, showed that dental pain in the last three months of 2011 reached 34%, which highlights the importance of health promotion actions, and not just treatment [10].

Although the association between sugar and dental caries is well documented [6], emerging evidence indicates that ultra-processed food also serves as a risk factor that contributes to an escalation of dental caries [7]. Its impact on self-reported dental pain, an important indicator of the quality of oral health, is still poorly explored.

Dental pain is an outcome of considerable clinical and epidemiological relevance, as it compromises physical and emotional well-being, school performance and quality of life of adolescents, making it an important marker of the need for oral health care [11-13]. Studying adolescents is essential, as this phase in life concentrates important behavioral changes and the consolidation of health habits, in addition to representing a critical period of vulnerability for the onset and worsening of oral problems [3,5,12]. In the context of the general population, dental pain is frequently used to indicate an unmet need for dental care.

The main cause of dental pain is untreated dental caries lesions, which are a persistent problem in several countries [11]. Dental caries is the most common non-communicable disease in the world and frequently causes pain and infection, in addition to impacting oral health throughout life [14]. Dental pain is one of the main reasons why adolescents seek urgent dental care in the Brazilian Unified Health System (*Sistema Único de Saúde*, SUS), which overburdens the public network and emphasizes the need for prevention strategies.

The 2023 National Oral Health Survey (*SB Brasil*) revealed that 20% of adolescents reported experiencing dental pain in the last six months [15], highlighting the magnitude of this problem in this age group. These facts underscore the importance of studying dental pain as a clinical and epidemiological outcome and highlight the gap in research linking risk factors to this subjective indicator of oral suffering among Brazilian adolescents.

This study aimed to help fill this gap by investigating association between ultra-processed food consumption and self-reported dental pain in a nationally representative sample of Brazilian school adolescents, based on data from the 2019 National School Health Survey, in order to provide support for public policies and effective prevention, which aligns with the objectives of the SUS.

The conceptual hypothesis of this study is that adolescents with higher ultra-processed food consumption have higher prevalence of dental pain, regardless of sociodemographic variables and use of dental services. As a secondary objective, we then sought to assess regional inequalities in this association.

## Methods

### Design

This is a cross-sectional study analyzing data obtained from the fourth edition of the National School Health Survey (*Pesquisa Nacional de Saúde do Escolar*, PeNSE), conducted in 2019, which presents a representative sample of the Brazilian school population aged between 13 and 17 years, this being the age group eligible for this study.

### Setting and participants

The PeNSE is a survey conducted by the Brazilian Institute of Geography and Statistics (IBGE) together with the Ministry of Health and the Ministry of Education and so far has been carried out in 2009, 2012, 2015 and 2019 [16,17]. The 2019 PeNSE employed a complex, multi-stage sampling design, with stratification by federative units, state capitals and other municipalities, which ensured national representativeness.

A two-stage cluster survey was used in order to comprise the sample, considering schools as the first stage and classes as the second stage. All students from the selected classes were invited to participate. Adolescents aged 13 to 17 enrolled at public and private schools, located in urban and rural areas throughout Brazil, were included. Additional information about the 2019 PeNSE can be found in the official publication [16].

Participant recruitment occurred through selected schools, with institutional authorization and voluntary student participation. Data collection took place in a school setting, in the classroom, using a self-administered electronic questionnaire on a mobile device provided by the IBGE. This strategy aimed to ensure standardization, confidentiality and anonymity of responses, as there was no direct intervention by researchers during the completion of the instrument.

Although no specific sample size calculation was performed for this study, the 2019 PeNSE survey sample size was large and built based on a complex and representative design, which guarantees high statistical power to detect the associations investigated. A total of 160,721 students took part in the survey, which, after a period of data review and consistency checks conducted by the IBGE, resulted in 125,123 valid answered questionnaires.

### Eligibility criteria

Only adolescents aged 13 to 17 years, participants in the 2019 PeNSE survey, who had valid information for the dependent variable assessed, i.e. self-reported dental pain, were included in the analysis.

### Variables of interest

The dependent variable was self-reported dental pain, measured by the question: “In the last six months, have you had dental pain that was not caused by use of dental braces?”, previously validated and used in national and international studies with adolescents, which demonstrated consistency as an epidemiological measure [11,12]. The response options included “yes” and “no”. Adolescents who answered “I don’t know/I don’t remember” were excluded from the analysis.

The independent variable and main form of exposure was ultra-processed food consumption. A combined approach was used for food classification, based on the *Dietary Guidelines for the Brazilian Population* [18] and the NOVA classification system [19]. In the context of the 2019 PeNSE, two aspects of eating habits were examined: habitual consumption and consumption on the immediately preceding day [20]. These variables were used in a complementary way, which enabled capturing both one-off consumption and the habitual pattern throughout the week.

Data on consumption in the last 24 hours was obtained from questions about intake of 13 specific food items: soda, processed juice, powdered drink mix, chocolate milk, flavored yogurt, processed snacks (e.g., packaged potato chips or crackers), processed sweet snacks (e.g., sweet biscuits, filled biscuits or packaged cake mix), processed desserts (e.g., chocolate, ice cream, jelly, pudding), processed meat products (e.g., sausage, mortadella or ham), processed bread (e.g., sliced ​​bread, hot dog buns or hamburger buns), margarine, processed sauces (e.g., mayonnaise or ketchup), and processed ready meals (e.g., instant noodles, packaged soups or frozen lasagna). Participants answered “yes” or “no” for each item.

Regarding consumption in the last seven days, participants were asked: “In the last seven days, on how many days did you eat sweet snacks, such as candies, sweets, chocolates, chewing gum, bonbons, lollipops, and others?”; “In the last seven days, on how many days did you drink soda?”; and “In the last seven days, on how many days did you eat at snack bars, hot dog stands, pizzerias or fast-food chains (fast-food meals)?”. Response options included: “I did not eat/drink any of these in the last seven days,” “I ate/drank them on one day,” “I ate/drank them on two days,” “I ate/drank them on three days,” “I ate/drank them on four days,” “I ate/drank them on five days,” “I ate/drank them on six days,” and “I ate/drank them every day.” For this analysis, weekly frequency was categorized as low (0-2 days), moderate (3-4 days) and high consumption (5-7 days). This categorization was adopted in order to assess a possible dose-response curve between consumption and dental pain.

Demographic and socioeconomic variables were included for characterization and adjustment of the analyses: sex (male or female), self-reported race/skin color (White, Black, Asian, mixed-race or Indigenous), age (13-15 years or 16-17 years), and maternal schooling (no schooling, elementary education, high school or complete higher education).

Use of dental services was assessed using the question: “In the last 12 months, how many times have you been to the dentist?”. Response options included: “Never in the last 12 months”, “Once”, “Twice”, “Three or more times”. The question was subsequently dichotomized into Visited the dentist in the last 12 months (yes/no).

### Data sources

The data used is publicly accessible, anonymized, and made available by IBGE through its institutional website (https://www.ibge.gov.br) [16].

### Statistical methods

Data collection was carried out using a validated electronic questionnaire, previously tested and standardized by the IBGE, which minimizes information bias. To reduce selection bias, a complex sampling design was used, ensuring national representativeness of adolescents from public and private schools.

Descriptive analysis was performed to present the absolute and relative frequencies of the study variables. Subsequently, bivariate analysis was conducted to explore associations between the variables of interest and the outcome (dental pain). Association between ultra-processed food consumption and dental pain was estimated using Poisson regression models with robust variance, with effect measures expressed as prevalence ratios (PR) and respective 95% confidence intervals (95%CI). Unadjusted models were estimated, followed by adjusted models.

Control for confounding was guided by a directed acyclic graph [21,22], which included sex, age group, race/skin color and maternal schooling as potential confounders. The “visit to the dentist” variable was taken into consideration in the model because it reflects the use of health services, with the possibility of being associated with the outcome even though it did not present a direct causal relationship with ultra-processed food consumption. Variables with a p-value<0.200 in the bivariate analysis were included in the multiple regression model using a stepwise procedure with backward elimination. Associations with a p-value<0.050 were considered statistically significant in the final model. Analysis of ultra-processed food consumption in different regions of Brazil was also performed, as well as its possible association with dental pain in adolescents. Considering the complex sampling design of the PeNSE, the Stata software version 17.0 (StataCorp.) svy command was used to ensure weighted estimates and robust confidence intervals.

It is important to highlight that the PeNSE did not include clinical dental examination, which made it impossible to directly assess the presence of caries lesions. Dental pain was used as a self-reported outcome, recognized in the literature as an indicator of the need for dental care in population studies.

## Results

The conceptual model that guided the selection of the adjustment variables included in the analyses is shown in Figure 1, which illustrates the relationship between ultra-processed food consumption, dental pain and potential confounders.

**Figure 1:**
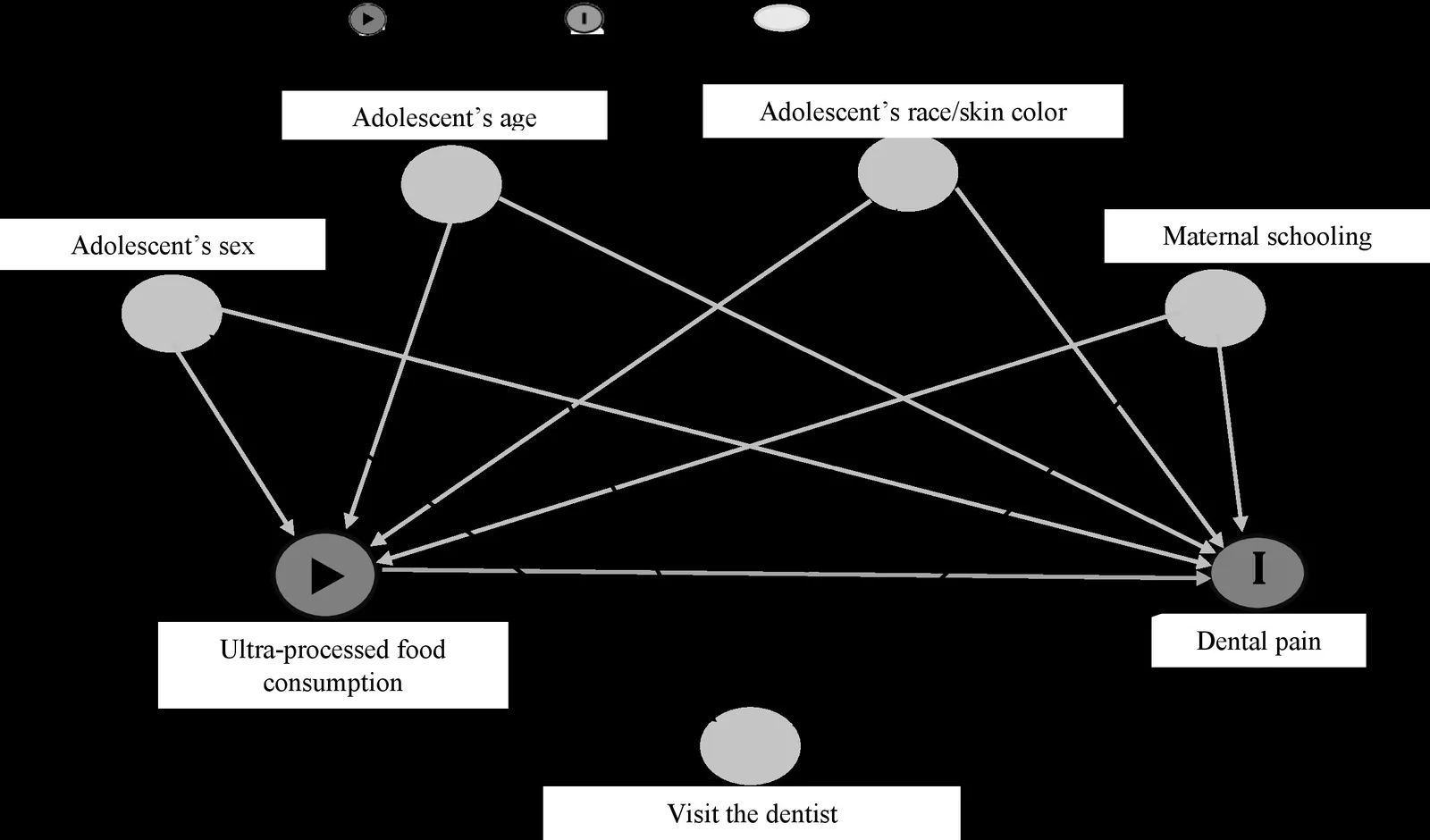
Relationships between ultra-processed food consumption, self-reported toothache and adjustment variables in Brazilian adolescents based on data from the National School Health Survey. Brazil, 2019

The analysis included 124,898 adolescents aged 13 to 17 years using PeNSE 2019 data. 66.0% were in the 13-15 age group and 34.0% were between 16 and 17 years old. A slight predominance of females (50.7%) was observed, and most participants self-identified as being of mixed race/skin color (43.6%) (Table 1).

**Table 1:** Proportion (%) and 95% confidence interval (95%CI) of the sample of Brazilian adolescents according to sociodemographic, oral health and ultra-processed food consumption characteristics, in addition to prevalence of self-reported dental pain, based on data from the National School Health Survey. Brazil, 2019 (n=124,898)

Study variables	Total	Presence of dental pain	
	n (%)	% (95%CI)	p-value
Sex			<0.001
Boys	61,462 (49.3)	18.5 (18.18; 18.91)	
Girls	63,148 (50.7)	23.8 (23.44; 24.22)	
**Age group **(years)			<0.001
13-15	82,389 (66.0)	20.3 (19.89; 20.66)	
16-17	42,509 (34.0)	23.9 (23.33; 24.42)	
**Self-reported race/skin color**			<0.001
White	47,498 (38.8)	19.4 (18.97; 19.80)	
Black	13,542 (11.1)	23.8 (22.97; 24.66)	
Asian	4,401 (3.6)	21.8 (20.41; 23.22)	
Mixed-race	53,413 (43.6)	22.1 (21.64; 22.48)	
Indigenous	3,512 (2.9)	21.6 (20.07; 23.17)	
**Maternal schooling**			<0.001
No schooling/Incomplete elementary education	18,121 (17.3)	25.5 (24.83; 26.29)	
Incomplete high school education	13,493 (12.8)	23.1 (22.30; 23.90)	
Incomplete higher education	33,475 (31.9)	21.5 (21.00; 22.01)	
Complete higher education	39,957 (38.0)	18.5 (18.03; 18.96)	
**Dental pain in the last six months**			
No	81,867 (78.5)	--	
Yes	22,355 (21.5)	--	
**Visited the dentist in the last 12 months**			0.0006
No	35,908 (28.9)	20.5 (20.00; 20.98)	
Yes	88,198 (71.1)	21.5 (21.17; 21.84)	
**Consumption of ultra-processed food in the last 24 hours**			0.0025
No	2,053 (1.6)	18.1 (16.27; 20.11)	
Yes	122,780 (98.4)	21.3 (20.98; 21.55)	
**Consumption of sweet food in the last seven days**			<0.001
Low (0-2 days)	50,460 (40.5)	18.8 (18.46; 19.25)	
Moderate (3-4 days)	33,127 (26.6)	20.6 (20.13; 21.13)	
High (5-7 days)	41,074 (32.9)	24.9 (24.35; 25.37)	
**Consumption of soda in the last seven days**			<0.001
Low (0-2 days)	77,137 (61.9)	19.7 (19.34; 20.02)	
Moderate (3-4 days)	27,457 (22.0)	22.8 (22.19; 23.37)	
High (5-7 days)	20,135 (16.1)	25.3 (24.63; 26.01)	
**Consumption of fast-food meals in the last seven days**			<0.001
Low (0-2 days)	102,362 (82.1)	20.7 (20.36; 20.96)	
Moderate (3-4 days)	14,469 (11.6)	23.2 (22.45; 24.04)	
High (5-7 days)	7,845 (6.3)	25.4 (24.31; 26.56)	

The total number of adolescents analyzed (n=124,898) changed according to the availability of information for each variable. The missing data were: sex (n=288), race/skin color (n=2,532), maternal schooling (n=19,852), dental pain (n=20,676), visited the dentist in the last 12 months (n=792), consumption of ultra-processed food in the last 24 hours (n=65), consumption of sweet food (n=237), consumption of soda (n=169) and consumption of fast-food meals (n=222). The analyses were performed using the data available for each variable.

Prevalence of self-reported dental pain in the last six months was 21.5%. Occurrence of pain was significantly higher among female adolescents (23.8%), adolescents aged 16-17 years (23.9%), those who self-identified as Black (23.8%), and among adolescents whose mothers had no schooling or incomplete elementary education (25.5%) (p-value<0.001). Regarding use of dental services, adolescents who reported visiting the dentist in the last 12 months had slightly higher prevalence of dental pain (21.5%) compared to those who had not had seen a dentist (20.5%) (p-value<0.001) (Table 1).

98.4% of the adolescents reported consuming ultra-processed food in the last 24 hours. Among these, prevalence of dental pain was higher (21.3%) compared to those who did not consume such foods (18.1%) (p-value 0.003). When considering consumption in the last seven days, a dose-response curve was observed, with a progressive increase in prevalence of dental pain as the frequency of consumption of sweet food, soda and fast-food meals increased. The highest prevalence rates were identified among adolescents classified in the high consumption category (5-7 days) for all food groups assessed (p-value<0.001) (Table 1).

When analyzing the frequency of ultra-processed food consumption among Brazilian adolescents by macro-region, we found that consumption in the last 24 hours was 97.9% in the Northern region, 98.6% in the Northeast, 98.5% in the Southeast, 98.4% in the South and 98.3% in the Midwest (p-value<0.001). Regarding weekly consumption, a higher proportion of high consumption of sweet food and soda was observed in the Midwest and Southeast regions, while the North and Northeast regions concentrated higher percentages of low consumption. Consumption of fast-food meals was predominantly low in all regions, although the Midwest, North and Northeast showed slightly higher percentages of high consumption (p-value<0.001) (Table 2).

**Table 2:** Proportion (%) of ultra-processed food consumption frequency among adolescents by Brazilian region based on data from the National School Health Survey. Brazil, 2019 (n=124,898)

Consumption of ultra-processed food	Frequency of ultra-processed food consumption by Brazilian macro-region					
	North	Northeast	Southeast	South	Midwest	p-value
	(%)	(%)	(%)	(%)	(%)	
**Consumption of ultra-processed food in the last 24 hours**						<0.001
No	2.1	1.4	1.5	1.6	1.7	
Yes	97.9	98.6	98.5	98.4	98.3	
**Consumption of sweet food in the last seven days**						<0.001
Low (0-2 days)	46.3	45.1	37.5	37.7	37.9	
Moderate (3-4 days)	24.6	24.7	27.6	27.8	26.5	
High (5-7 days)	29.1	30.2	34.9	34.5	35.6	
**Consumption of soda in the last seven days**						<0.001
Low (0-2 days)	64.7	66.3	59.6	61.5	55.1	
Moderate (3-4 days)	20.4	20.4	22.6	21.8	23.7	
High (5-7 days)	14.9	13.3	17.8	16.7	21.2	
**Consumption of fast-food meals in the last seven days**						<0.001
Low (0-2 days)	82.7	82.4	83.4	86.1	81.1	
Moderate (3-4 days)	10.7	11.0	11.6	9.8	12.2	
High (5-7 days)	6.6	6.6	5.0	4.1	6.7	

In the analysis adjusted for sex, age, race/skin color, maternal schooling and dentist visit, we found that ultra-processed food consumption in the last 24 hours maintained significant association with higher prevalence of dental pain (PR 1.27; 95%CI 1.10; 1.46). This association refers to consumption of some specific groups of ultra-processed food assessed in the study. Frequent consumption in the last seven days was also consistently associated with dental pain. Adolescents with high consumption of sweet food presented higher prevalence of dental pain (PR 1.27; 95%CI 1.22; 1.31) when compared to those with low consumption. A similar pattern was observed for consumption of soda (PR 1.28; 95%CI 1.23; 1.32) and fast-food meals (PR 1.22; 95%CI 1.15; 1.29), which revealed a dose-response curve between higher frequency of consumption of these foods and occurrence of dental pain (Table 3).

**Table 3:** Prevalence ratios (PR) and unadjusted and adjusted 95% confidence intervals (95%CI) of self-reported dental pain according to National School Health Survey data variables. Brazil, 2019 (n=124,898)

Consumption of ultra-processed food	Dental pain		
	Model 1^a^ PR (95%CI)	Model 2^b^ PR (95%CI)	Model 3^c^ PR (95%CI)
**Consumption of ultra-processed food in the last 24 hours**			
No	Reference	Reference	Reference
Yes	1.17 (1.05; 1.31)	1.26 (1.10; 1.44)	1.27 (1.10; 1.46)
**Consumption of sweet food in the last seven days**			
Low (0-2 days)	Reference	Reference	Reference
Moderate (3-4 days)	1.09 (1.06; 1.13)	1.10 (1.06; 1.14)	1.09 (1.05; 1.13)
High (5-7 days)	1.32 (1.28; 1.36)	1.28 (1.23; 1.32)	1.27 (1.22; 1.31)
**Consumption of soda in the last seven days**			
Low (0-2 days)	Reference	Reference	Reference
Moderate (3-4 days)	1.16 (1.12; 1.19)	1.17 (1.13; 1.21)	1.16 (1.12; 1.21)
High (5-7 days)	1.29 (1.24; 1.33)	1.28 (1.23; 1.33)	1.28 (1.23; 1.32)
**Consumption of fast-food meals in the last seven days**			
Low (0-2 days)	Reference	Reference	Reference
Moderate (3-4 days)	1.12 (1.08; 1.17)	1.12 (1.08; 1.17)	1.12 (1.08; 1.17)
High (5-7 days)	1.23 (1.17; 1.29)	1.22 (1.15; 1.29)	1.22 (1.15; 1.29)

^a^unadjusted; ^b^adjustad for sex, age, self-reported race/skin color, maternal schooling; ^c^modelo 2 adjusted for visit to the dentist.

In the analysis stratified by Brazilian regions, an increase in dental pain prevalence was observed as the frequency of consumption of sweet food, soda and fast-food meals increased in all regions of the country. Among adolescents with low sweet food consumption, prevalence of pain ranged from 16.7% in the Southern region to 21.2% in the Northern region. In the high-consumption category, the percentages ranged from 23.0% in the South to 27.2% in the North. A similar pattern was observed for soda consumption, with lower prevalence rates of pain among adolescents with low consumption in the Southern region (17.6%) and higher prevalence rates among those with high consumption in the Northern region (27.5%). Regarding fast-food meal consumption, prevalence of dental pain progressively increased in all regions as the frequency of consumption increased. Among adolescents with high consumption, the highest prevalence rates were observed in the Southeast (26.7%) and Northeast (25.6%), while in the South prevalence was 24.8% (Table 4).

**Table 4:** Prevalence (%) and 95% confidence intervals (95%CI) of self-reported dental pain in adolescents according to frequency of consumption of ultra-processed food, sweet food, soda and fast-food meals by Brazilian region based on data from the National School Health Survey. Brazil, 2019 (n=124,898)

Consumption of ultra-processed food	Dental pain by Brazilian region				
	North % (95%CI)	Northeast % (95%CI)	Southeast % (95%CI)	South % (95%CI)	Midwest % (95%CI)
**Consumption of sweet food in the last seven days**					
Low (0-2 days)	21.2 (20.3; 22.0)	18.8 (18.1; 19.4)	17.3 (16.3; 18.4)	16.7 (15.6; 17.8)	18.9 (17.7; 20.2)
Moderate (3-4 days)	23.0 (21.9; 24.1)	20.7 (19.8; 21.6)	19.3 (18.2; 20.5)	19.3 (18.0; 20.6)	20.2 (18.8; 21.8)
High (5-7 days)	27.2 (26.1; 28.3)	24.9 (24.0; 25.7)	23.8 (22.6; 25.0)	23.0 (21.7; 24.4)	25.0 (23.6; 26.5)
**Consumption of soda in the last seven days**					
Low (0-2 days)	22.0 (21.3; 22.7)	19.8 (19.2; 20.3)	18.4 (17.5; 19.2)	17.6 (16.6; 18.5)	19.4 (18.4; 20.4)
Moderate (3-4 days)	24.7 (23.4; 26.1)	22.6 (21.6; 23.6)	21.7 (20.3; 23.0)	21.6 (20.0; 23.3)	23.0 (21.5; 24.5)
High (5-7 days)	27.5 (25.9; 29.0)	25.4 (24.0; 26.7)	24.2 (22.7; 25.7)	24.4 (22.6; 26.3)	24.9 (23.4; 26.5)
**Consumption of fast-food meals in the last seven days**					
Low (0-2 days)	23.1 (22.4; 23.7)	20.4 (19.9; 20.9)	19.6 (18.8; 20.3)	19.1 (18.3; 19.9)	20.7 (19.9; 21.5)
Moderate (3-4 days)	24.8 (23.1; 26.4)	23.3 (22.0; 24.7)	21.3 (19.6; 23.2)	21.4 (19.2; 23.9)	24.7 (22.6; 27.0)
High (5-7 days)	25.0 (23.0; 27.3)	25.6 (23.7; 27.5)	26.7 (24.0; 29.7)	24.8 (21.1; 29.0)	24.5 (21.8; 27.4)

## Discussion

This study identified that higher frequency of ultra-processed food consumption is associated with higher prevalence of dental pain in Brazilian adolescents, even after adjusting for sociodemographic variables and use of dental services. High consumption of sweet food, soda and fast-food meals showed consistent association with the outcome, demonstrating a dose-response curve. These findings confirm the study’s initial hypothesis and reinforce the relevance of diet as a determinant of oral health in adolescence, a phase marked by the consolidation of risky habits and behaviors.

The results should be interpreted considering that the population studied comprised adolescents between 13 and 17 years old, an age group characterized by high exposure to ultra-processed food and greater food autonomy, which may explain the high prevalence of consumption observed. Due to their composition rich in free sugars, additives and fermentable carbohydrates, these products favor the formation and maturation of cariogenic biofilm, which contributes to the development and progression of dental caries, the main cause of pain in this age group [19,23,24]. Previous studies have already indicated that high ultra-processed food consumption increases the risk of caries in children and adolescents [5,7], and the results of this study expanded this evidence by demonstrating that they are also related to reported pain, an important marker of quality of life [11,13]. Dental pain, unlike caries as a clinical condition, is a direct manifestation of the suffering and functional limitation caused by oral disease. Although not all caries lesions progress to pain, their occurrence generally reflects more advanced stages of the disease, when there is dentin or pulp involvement, which triggers inflammation and painful symptoms that affect the physical and psychological well-being of adolescents [11-13]. Pain proves to be the most immediate indicator of the need for treatment and impact on quality of life.

Regional analysis showed that the association between higher consumption of sweet food, soda and fast-food meals and higher prevalence of dental pain was present in all the country’s macro-regions, although with variations in the magnitude of the effect. The North and Southeast regions showed higher prevalence rates of dental pain associated with high ultra-processed food consumption, while the Southern region, in general, showed the lowest percentages. These contrasts possibly reflect regional inequalities related to dietary patterns, access to fresh and minimally processed foods, and the organization of health services, which highlights the importance of public policies sensitive to territorial specificities [25,26].

Female adolescents, older adolescents and those with lower maternal schooling showed higher prevalence of dental pain, a consistent and socially unequal pattern as per findings from other national [12] and international [3] studies. The association observed with dental visits may reflect seeking care due to pain and limitations in the effectiveness of the care provided, which highlights the relevance of preventive actions [14]. Maternal schooling, frequently used as an indirect marker of socioeconomic status, proved to be an important indicator of inequality, since evidence suggests that adolescents from families with lower maternal schooling and poorer socioeconomic conditions tend to consume more ultra-processed food [25], which may explain some of the inequalities observed in the prevalence of pain. These results emphasize that dental pain should not be understood only as a clinical problem, but also as an expression of social inequities.

The findings of this study are consistent with international recommendations, which have advised limiting consumption of ultra-processed and sugary foods to less than 5% of total daily caloric intake [14,27]. The high frequency of consumption identified, almost universal among adolescents, reinforces the need for educational and regulatory strategies that encourage healthier food choices, with positive repercussions on oral health and prevention of chronic non-communicable diseases [1,28]. The implementation of healthy school environments, as established by Decree No. 11,821/2023, which establishes guidelines for promoting healthy eating in educational institutions, is an important step for supporting food policies that protect the health of the adolescent population and promote intersectoral prevention practices [29].

Standing out among the study’s limitations is the use of self-reported measurements for both pain and food consumption, which are subject to memory and perception bias. The absence of clinical dental examinations made it impossible to confirm the presence of caries lesions, the main cause of dental pain. The food questionnaire, in turn, although validated, does not enable quantifying intake or assessing the detailed composition of the products consumed. Nevertheless, several measures were taken to minimize possible biases and strengthen the validity of the findings, such as the use of standardized instruments validated by the IBGE, the complex sampling design, which ensured national representativeness, and the adjustment of analyses for potential confounding variables, which increased the robustness and validity of the findings.

High consumption of ultra-processed food has been consistently associated with higher prevalence of dental pain in Brazilian adolescents. The results highlighted the need for health promotion strategies and public policies that reduce consumption of ultra-processed food during adolescence, contributing to preventing dental pain and improving quality of life. Future longitudinal studies including clinical examinations and multilevel analyses could deepen the understanding of the causal relationships between ultra-processed food consumption, dental pain and health inequities.

## Data Availability

The data used in this article are available at: https://osf.io/gxqdt/.
