# Supplementary material for: Ultra-processed food consumption and dental pain in adolescents: a descriptive analysis, Brazil, 2019
Source: Epidemiol Serv Saude. 2026 Jul 6;35:e20250508. doi: 10.1590/S2237-96222026v35e20250508.en (PMC13686385; doi:10.1590/S2237-96222026v35e20250508.en)
Supplement: Translation

# Translation: Consumo de alimentos ultraprocessados e dor dentária em adolescentes: análise descritiva, Brasil, 2019

**DOI:** 10.1590/S2237-96222026v35e20250508.pt

**Authors:** Nathalia Ribeiro Jorge da Silva-Garcia, Sarah Arangurem Karam, Eduardo Dickie de Castilhos, Mariana Gonzalez Cademartori

**Affiliations:** 1 Universidade Federal de Pelotas Programa de Pós-Graduação em Odontologia Pelotas RS Brasil Universidade Federal de Pelotas, Programa de Pós-Graduação em Odontologia, Pelotas, RS, Brasil; 2 Universidade Católica de Pelotas Programa de Pós-Graduação em Saúde no Ciclo Vital Pelotas RS Brasil Universidade Católica de Pelotas, Programa de Pós-Graduação em Saúde no Ciclo Vital, Pelotas, RS, Brasil

**Keywords:** Dor Dentária, Consumo Alimentar, Adolescentes, Alimentos Ultraprocessados, Vigilância em Saúde Pública

## Abstract

**Objetivo:**

Analisar a associação entre o consumo de alimentos ultraprocessados e o relato de dor dentária em adolescentes brasileiros.

**Métodos:**

Estudo transversal com dados da Pesquisa Nacional de Saúde do Escolar de 2019 com adolescentes de 13 a 17 anos. O desfecho foi a dor dentária autorreferida nos últimos seis meses. O consumo de alimentos ultraprocessados foi avaliado a partir da frequência de ingestão nas últimas 24 horas e nos últimos sete dias, segundo a classificação NOVA, com a consideração do consumo de doces, refrigerantes e refeições do tipo fast-food categorizado como baixo (0-2 dias), moderado (3-4 dias) ou alto (5-7 dias). As associações foram estimadas por meio de razões de prevalência (RP) ajustadas por sexo, idade, raça/cor da pele, escolaridade materna e visita ao dentista.

**Resultados:**

Dos 124.898 adolescentes analisados, 21,5% relataram dor dentária. 98,4% consumiram alimento ultraprocessado nas últimas 24 horas. Observou-se baixo consumo de doces (40,5%), refrigerantes (61,9%) e refeições do tipo fast-food (82,1%) quanto à frequência semanal. Na análise ajustada, a prevalência de dor dentária foi maior entre adolescentes com alto consumo de doces (RP 1,27; IC95% 1,22; 1,31), refrigerantes (RP 1,28; IC95% 1,23; 1,32) e refeições do tipo fast-food (RP 1,22; IC95% 1,15; 1,29) na última semana, comparados àqueles com baixo consumo.

**Conclusão:**

Maior frequência de consumo de alimentos ultraprocessados está associada a maior prevalência de dor dentária entre adolescentes brasileiros, o que reforça a relevância de estratégias intersetoriais de promoção da alimentação saudável e de prevenção em saúde bucal nessa faixa etária.

**Aspectos éticos**

Esta pesquisa utilizou bancos de dados de domínio público e anonimizados.

## Introdução

O aumento global do consumo de alimentos ultraprocessados têm se mostrado um importante fator de risco para doenças crônicas não transmissíveis [1]. No reconhecimento de que hábitos alimentares inadequados representam uma preocupação significativa de saúde pública e afetam adversamente vários resultados de saúde, a Organização Mundial da Saúde tem orientado a redução do consumo desses produtos, especialmente por crianças e adolescentes, períodos críticos para o estabelecimento de padrões alimentares de longo prazo [2,3]. Pesquisas focadas nessas fases do desenvolvimento assumem, assim, suma importância [4,5].

Estudos brasileiros mostram elevada prevalência de dor dentária em adolescentes: 19% de adolescentes de 12 anos em Minas Gerais [8] e 30% de adolescentes entre 15 e 19 anos no Sul do Brasil [9]. Outra pesquisa, no contexto da Estratégia de Saúde da Família de Manaus, mostrou que a dor dentária nos últimos três meses de 2011 chegou a 34%, o que destaca a importância de ações de promoção da saúde, não apenas de tratamento [10]. 

Embora a associação entre açúcar e cárie dentária seja bem documentada [6], evidências emergentes indicam que alimentos ultraprocessados também servem como fatores de risco que contribuem para uma escalada da cárie dentária [7]. Seu impacto sobre a dor dentária autorreferida, importante indicador da qualidade de vida oral, ainda é pouco explorado. 

A dor dentária é um desfecho de grande relevância clínica e epidemiológica, pois compromete o bem-estar físico e emocional, o desempenho escolar e a qualidade de vida de adolescentes, o que a faz ser um marcador importante da necessidade de cuidados em saúde bucal [11-13]. Estudar adolescentes é essencial, pois essa fase concentra importantes mudanças comportamentais e consolidação de hábitos de saúde, além de representar período crítico de vulnerabilidade para o início e o agravamento de problemas bucais [3,5,12]. No contexto populacional, a dor dentária é frequentemente utilizada para indicar a necessidade não atendida de cuidado odontológico.

A principal causa para a ocorrência de dor dentária são as lesões de cárie dentária não tratadas, que se configuram como problema persistente em vários países [11]. A cárie dentária é a doença não transmissível mais comum no mundo e frequentemente causa dor e infecção, além de impactar a saúde bucal ao longo da vida [14]. A dor dentária é um dos principais motivos que levam adolescentes a buscar atendimento odontológico de urgência no Sistema Único de Saúde (SUS), o que sobrecarrega a rede pública e reforça a necessidade de estratégias preventivas.

A pesquisa nacional SB Brasil 2023 revelou que 20% dos adolescentes relataram sentir dor de dente nos últimos seis meses [15], o que evidencia a magnitude desse problema nessa faixa etária. Esses fatos ressaltam a importância de estudar a dor dentária como desfecho clínico e epidemiológico e reforça a lacuna existente em investigações que vinculem fatores de risco a esse indicador subjetivo de sofrimento oral entre adolescentes brasileiros. 

Este estudo propôs-se a auxiliar no preenchimento dessa lacuna a partir da investigação da associação entre o consumo de alimentos ultraprocessados e a dor dentária autorreferida em amostra nacionalmente representativa de adolescentes escolares brasileiros, com base em dados da Pesquisa Nacional de Saúde do Escolar de 2019, a fim de fornecer subsídios para políticas públicas e prevenção eficaz, o que se alinha aos objetivos do SUS.

A hipótese conceitual deste estudo é a de que adolescentes com maior consumo de alimentos ultraprocessados apresentam maior prevalência de dor dentária, independentemente de variáveis sociodemográficas e de uso de serviços odontológicos. Como objetivo secundário, buscou-se, então, avaliar as desigualdades regionais nessa associação.

## Métodos

### Delineamento do estudo

Trata-se de estudo transversal com análise de dados obtidos a partir da quarta edição da Pesquisa Nacional de Saúde do Escolar (PeNSE), realizada em 2019, que apresenta uma amostra representativa da população escolar brasileira com idade entre 13 e 17 anos, faixa etária elegível para este estudo.

### Contexto e participantes

A PeNSE é um estudo realizado pelo Instituto Brasileiro de Geografia e Estatística (IBGE) juntamente com o Ministério da Saúde e o Ministério da Educação e possui quatro edições, realizadas em 2009, 2012, 2015 e 2019 [16,17]. A PeNSE 2019 empreendeu um desenho amostral complexo, em múltiplos estágios, com estratificação por unidades da federação, capitais e demais municípios, o que garantiu representatividade nacional. 

Para a composição da amostra, foi utilizada uma sondagem por conglomerados em dois estágios, com consideração de escolas como primeiro estágio e de turmas como segundo estágio. Todos os estudantes das turmas sorteadas foram convidados a participar. Foram incluídos adolescentes de 13 a 17 anos matriculados em escolas públicas e em privadas, situadas em áreas urbanas e em rurais de todo o território brasileiro. Informações adicionais sobre a PeNSE 2019 podem ser encontradas em publicação oficial [16].

O recrutamento dos participantes ocorreu por meio das escolas selecionadas, com autorização institucional e participação voluntária dos estudantes. A coleta de dados foi realizada em ambiente escolar, sala de aula, com utilização de questionário eletrônico autopreenchido em dispositivo móvel fornecido pelo IBGE. Essa estratégia visou garantir padronização, sigilo e anonimato das respostas, uma vez que não houve intervenção direta dos pesquisadores durante o preenchimento do instrumento. 

Embora não tenha sido realizado cálculo amostral específico para este estudo, a amostra da PeNSE 2019 foi de grande magnitude e construída com base em delineamento complexo e representativo, o que garante elevado poder estatístico para detectar as associações investigadas. Participaram da pesquisa 160.721 alunos, que, após período de crítica e consistência dos dados conduzido pelo IBGE, resultaram em 125.123 questionários válidos.

### Critérios de elegibilidade

Foram incluídos na análise apenas adolescentes com idade entre 13 e 17 anos, participantes da PeNSE 2019, que apresentavam informações válidas para a variável dependente avaliada, dor dentária autorreferida.

### Variáveis de interesse

A variável dependente foi dor dentária autorreferida, mensurada pela pergunta: “Nos últimos seis meses, você teve dor de dente que não foi causada pelo uso de aparelho ortodôntico?”, previamente validada e empregada em estudos nacionais e internacionais com adolescentes, o que demonstrou consistência como medida epidemiológica [11,12]. As opções de resposta incluíam “sim” e “não”. Os adolescentes que responderam “não sei/não me lembro” foram excluídos da análise.

A variável independente e principal exposição foi o consumo de alimentos ultraprocessados. Para a classificação dos alimentos, foi utilizada uma abordagem combinada, baseada no *Guia Alimentar para a População Brasileira* [18] e no sistema de classificação NOVA [19]. No contexto da PeNSE 2019, foram examinados dois aspectos dos hábitos alimentares: o consumo habitual e o consumo no dia imediatamente anterior [20]. Essas variáveis foram utilizadas de forma complementar, o que permitiu captar o consumo pontual e o padrão habitual ao longo da semana.

Os dados sobre o consumo nas últimas 24 horas foram obtidos a partir de perguntas sobre a ingestão de 13 itens alimentares específicos: refrigerante, suco industrializado, refresco em pó, bebida achocolatada, iogurte saborizado, salgadinhos industrializados (ex.: batata frita de pacote ou biscoitos salgados), lanches doces industrializados (ex.: biscoito doce, biscoito recheado ou bolo de pacote), sobremesas industrializadas (ex.: chocolate, sorvete, gelatina, pudim), produtos de carne processada (ex.: salsicha, mortadela ou presunto), pães industrializados (ex.: pão de forma, pão de cachorro-quente ou pão de hambúrguer), margarina, molhos industrializados (ex.: maionese ou ketchup) e refeições prontas industrializadas (ex.: macarrão instantâneo, sopas de pacote ou lasanha congelada). Os participantes responderam “sim” ou “não” para cada item.

Com relação ao consumo nos últimos sete dias, os participantes foram questionados: “Nos últimos sete dias, em quantos dias você consumiu lanches doces, como balas, confeitos, chocolates, chicletes, bombons, pirulitos e outros?”; “Nos últimos sete dias, em quantos dias você tomou refrigerante?” e “Nos últimos sete dias, em quantos dias você comeu em lanchonetes, carrinhos de cachorro-quente, pizzarias ou redes de fast-food (refeições do tipo fast-food)?”. As opções de resposta incluíam: “Não consumi nos últimos sete dias”, “Consumi em um dia”, “Consumi em dois dias”, “Consumi em três dias”, “Consumi em quatro dias”, “Consumi em cinco dias”, “Consumi em seis dias” e “Consumi todos os dias”. Para essa análise, a frequência semanal foi categorizada como baixo (0-2 dias), moderado (3-4 dias) e alto consumo (5-7 dias). Essa categorização foi adotada com o objetivo de avaliar possível gradiente dose-resposta entre consumo e dor dentária.

Foram incluídas variáveis demográficas e socioeconômicas para caracterização e ajuste das análises: sexo (masculino ou feminino), raça/cor da pele autodeclarada (branca, preta, amarela, parda ou indígena), idade (13-15 anos ou 16-17 anos) e escolaridade materna (nenhuma escolaridade, ensino fundamental, ensino médio ou ensino superior completo).

A utilização de serviços odontológicos foi avaliada por meio da pergunta: “Nos últimos 12 meses, quantas vezes você foi ao dentista?”. As opções de resposta incluíam: “Nenhuma vez nos últimos 12 meses”, “Uma vez”, “Duas vezes”, “Três ou mais vezes”. A pergunta foi posteriormente dicotomizada em visita ao dentista no último ano (sim/não). 

### Fontes de dados

Os dados utilizados são de acesso público, anonimizados e disponibilizados pelo IBGE por meio de sítio eletrônico institucional (https://www.ibge.gov.br) [16].

### Métodos estatísticos

A coleta de dados foi realizada com questionário eletrônico validado, previamente testado e padronizado pelo IBGE, o que minimiza o viés de informação. Para reduzir o viés de seleção, foi utilizado delineamento amostral complexo, o que garantiu representatividade nacional de adolescentes de escolas públicas e privadas. 

Foi realizada análise descritiva para apresentação das frequências absolutas e relativas das variáveis do estudo. Em seguida, procedeu-se à análise bivariada para explorar associações entre as variáveis de interesse e o desfecho (dor dentária). A associação entre o consumo de alimentos ultraprocessados e a dor dentária foi estimada por meio de modelos de regressão de Poisson com variância robusta, com as medidas de efeito expressas como razão de prevalência (RP) e respectivos intervalos de confiança de 95% (IC95%). Foram estimados modelos não ajustados, seguidos de modelos ajustados.

O controle de confundimento foi orientado por gráfico acíclico direcionado [21,22], que incluiu sexo, faixa etária, raça/cor da pele e escolaridade materna como potenciais confundidores. A variável “visita ao dentista” foi considerada no modelo por refletir o uso de serviços de saúde, com possibilidade de estar associada ao desfecho ainda que não apresentasse relação causal direta com o consumo de alimentos ultraprocessados. As variáveis com p-valor<0,200 na análise bivariada foram inseridas no modelo de regressão múltipla por procedimento *stepwise *com eliminação retroativa. Associações com p-valor<0,050 foram consideradas estatisticamente significativas no modelo final. Foi realizada também análise do consumo de alimentos ultraprocessados nas diferentes regiões do Brasil, bem como sua possível associação com dor dentária em adolescentes. Com a consideração do delineamento amostral complexo da PeNSE, foi utilizado o comando svy no software Stata versão 17.0 (StataCorp.) para garantir estimativas ponderadas e intervalos de confiança robustos.

Cabe destacar que a PeNSE não incluiu exame clínico odontológico, o que impossibilitou a avaliação direta sobre a presença de lesões de cárie. A dor dentária foi utilizada como desfecho autorreferido, reconhecida na literatura como indicador de necessidade de cuidado odontológico em estudos populacionais.

## Resultados

O modelo conceitual que orientou a seleção das variáveis de ajuste incluídas nas análises foi apresentado na Figura 1, que ilustrou a relação entre consumo de alimentos ultraprocessados, dor dentária e potenciais confundidores. 

**Figura 1:** Relações entre consumo de alimentos ultraprocessados, dor dentária autorreferida e variáveis de ajuste em adolescentes brasileiros a partir de dados da Pesquisa Nacional de Saúde do Escolar. Brasil, 2019

Participaram da análise 124.898 adolescentes com idade entre 13 e 17 anos provenientes de dados da PeNSE 2019. 66,0% encontravam-se na faixa etária de 13 a 15 anos e 34,0% tinham entre 16 e 17 anos. Observou-se leve predominância do sexo feminino (50,7%) e a maior parte dos participantes se autodeclarou de raça/cor da pele parda (43,6%) (Tabela 1). 

**Tabela 1:** Proporção (%) e intervalo de confiança de 95% (IC95%) da amostra segundo características sociodemográficas, de saúde bucal e de consumo alimentar de alimentos ultraprocessados por adolescentes brasileiros, além da prevalência de dor dentária autorreferida, a partir de dados da Pesquisa Nacional de Saúde do Escolar. Brasil, 2019 (n=124.898)

Variáveis do estudo	Total	Presença de dor dentária	
	n (%)	% (IC95%)	p-valor
Sexo			<0,001
Meninos	61.462 (49,3)	18,5 (18,18; 18,91)	
Meninas	63.148 (50,7)	23,8 (23,44; 24,22)	
**Faixa etária **(anos)			<0,001
13-15	82.389 (66,0)	20,3 (19,89; 20,66)	
16-17	42.509 (34,0)	23,9 (23,33; 24,42)	
**Raça/cor da pele autodeclarada**			<0,001
Branca	47.498 (38,8)	19,4 (18,97; 19,80)	
Preta	13.542 (11,1)	23,8 (22,97; 24,66)	
Amarela	4.401 (3,6)	21,8 (20,41; 23,22)	
Parda	53.413 (43,6)	22,1 (21,64; 22,48)	
Indígena	3.512 (2,9)	21,6 (20,07; 23,17)	
**Escolaridade materna**			<0,001
Sem escolaridade/ensino fundamental incompleto	18.121 (17,3)	25,5 (24,83; 26,29)	
Ensino médio incompleto	13.493 (12,8)	23,1 (22,30; 23,90)	
Ensino superior incompleto	33.475 (31,9)	21,5 (21,00; 22,01)	
Ensino superior completo	39.957 (38,0)	18,5 (18,03; 18,96)	
**Dor de dente nos últimos seis meses**			
Não	81.867 (78,5)	--	
Sim	22.355 (21,5)	--	
**Visita ao dentista no último ano**			0,0006
Não	35.908 (28,9)	20,5 (20,00; 20,98)	
Sim	88.198 (71,1)	21,5 (21,17; 21,84)	
**Consumo de alimentos ultraprocessados nas últimas 24 horas**			0,0025
Não	2.053 (1,6)	18,1 (16,27; 20,11)	
Sim	122.780 (98,4)	21,3 (20,98; 21,55)	
**Consumo de doces nos últimos sete dias**			<0,001
Baixo (0-2 dias)	50.460 (40.5)	18,8 (18,46; 19,25)	
Moderado (3-4 dias)	33.127 (26,6)	20,6 (20,13; 21,13)	
Alto (5-7 dias)	41.074 (32,9)	24,9 (24,35; 25,37)	
**Consumo de refrigerantes nos últimos sete dias**			<0,001
Baixo (0-2 dias)	77.137 (61.9)	19,7 (19,34; 20,02)	
Moderado (3-4 dias)	27.457 (22.0)	22,8 (22,19; 23,37)	
Alto (5-7 dias)	20.135 (16,1)	25,3 (24,63; 26,01)	
**Consumo de refeições do tipo fast-food nos últimos sete dias**			<0,001
Baixo (0-2 dias)	102.362 (82,1)	20,7 (20,36; 20,96)	
Moderado (3-4 dias)	14.469 (11,6)	23,2 (22,45; 24,04)	
Alto (5-7 dias)	7.845 (6,3)	25,4 (24,31; 26,56)	

O número total de adolescentes analisados (n=124.898) se alterou conforme a disponibilidade de informações em cada variável. Os dados faltantes foram: sexo (n=288), raça/cor da pele (n=2.532), escolaridade materna (n=19.852), dor dentária (n=20.676), visita ao dentista no último ano (n=792), consumo de alimentos ultraprocessados nas últimas 24 horas (n=65), consumo de doces (n=237), consumo de refrigerantes (n=169) e consumo de refeições do tipo fast-food (n=222). As análises foram realizadas com os dados válidos disponíveis para cada variável.

A prevalência de dor dentária autorreferida nos últimos seis meses foi de 21,5%. A ocorrência de dor foi significativamente maior entre adolescente do sexo feminino (23,8%), adolescentes entre 16 e 17 anos (23,9%), aqueles que se autodeclararam de raça/cor da pele preta (23,8%) e entre os adolescentes cujas mães não possuíam escolaridade ou apresentavam ensino fundamental incompleto (25,5%) (p-valor<0,001). Quanto ao uso de serviços odontológicos, adolescentes que relataram visita ao dentista no último ano apresentaram prevalência ligeiramente maior de dor dentária (21,5%) quando comparados àqueles que não realizaram consulta (20,5%) (p-valor<0,001) (Tabela 1). 

98,4% dos adolescentes relataram consumo de alimentos ultraprocessados nas últimas 24 horas. Entre esses, a prevalência de dor dentária foi maior (21,3%) em comparação aos que não consumiram tais alimentos (18,1%) (p-valor 0,003). Ao considerar o consumo nos últimos sete dias, observou-se gradiente dose-resposta, com aumento progressivo da prevalência de dor dentária conforme a frequência de consumo de doces, refrigerantes e refeições do tipo fast-food foi maior. As prevalências mais altas foram identificadas entre os adolescentes classificados na categoria de alto consumo (5-7 dias) para todos os grupos alimentares avaliados (p-valor<0,001) (Tabela 1).

Na análise da frequência do consumo de alimentos ultraprocessados entre adolescentes brasileiros segundo macrorregiões, constatou-se que o consumo nas últimas 24 horas foi de 97,9% na região Norte, 98,6% no Nordeste, 98,5% no Sudeste, 98,4% no Sul e 98,3% no Centro-Oeste (p-valor<0,001). Em relação ao consumo semanal, observou-se maior proporção de alto consumo de doces e refrigerantes nas regiões Centro-Oeste e Sudeste, enquanto as regiões Norte e Nordeste concentraram maiores percentuais de baixo consumo. O consumo de refeições do tipo fast-food foi predominantemente baixo em todas as regiões, embora Centro-Oeste, Norte e Nordeste tenham apresentado percentuais ligeiramente mais elevados de alto consumo (p-valor<0,001) (Tabela 2).

**Tabela 2:** Proporção (%) da frequência do consumo de alimentos ultraprocessados entre adolescentes por região brasileira a partir de dados da Pesquisa Nacional de Saúde do Escolar. Brasil, 2019 (n=124.898)

Consumo de alimentos ultraprocessados	Frequência do consumo de alimentos ultraprocessados por macrorregião brasileira					
	Norte	Nordeste	Sudeste	Sul	Centro-Oeste	p-valor
	(%)	(%)	(%)	(%)	(%)	
**Consumo de alimentos ultraprocessados nas últimas 24 horas**						<0,001
Não	2,1	1,4	1,5	1,6	1,7	
Sim	97,9	98,6	98,5	98,4	98,3	
**Consumo de doces nos últimos sete dias**						<0,001
Baixo (0-2 dias)	46,3	45,1	37,5	37,7	37,9	
Moderado (3-4 dias)	24,6	24,7	27,6	27,8	26,5	
Alto (5-7 dias)	29,1	30,2	34,9	34,5	35,6	
**Consumo de refrigerantes nos últimos sete dias**						<0,001
Baixo (0-2 dias)	64,7	66,3	59,6	61,5	55,1	
Moderado (3-4 dias)	20,4	20,4	22,6	21,8	23,7	
Alto (5-7 dias)	14,9	13,3	17,8	16,7	21,2	
**Consumo de refeições do tipo fast-food nos últimos sete dias**						<0,001
Baixo (0-2 dias)	82,7	82,4	83,4	86,1	81,1	
Moderado (3-4 dias)	10,7	11,0	11,6	9,8	12,2	
Alto (5-7 dias)	6,6	6,6	5,0	4,1	6,7	

Na análise ajustada por sexo, idade, raça/cor de pele, escolaridade materna e visita ao dentista, verificou-se que o consumo de alimentos ultraprocessados nas últimas 24 horas manteve associação significativa com maior prevalência de dor dentária (RP 1,27; IC95% 1,10; 1,46). Tal associação refere-se ao consumo de alguns grupos específicos de alimentos ultraprocessados avaliados no estudo. O consumo frequente nos últimos sete dias, da mesma forma, mostrou-se consistentemente associado à dor dentária. Adolescentes com alto consumo de doces apresentaram maior prevalência de dor dentária (RP 1,27; IC95% 1,22; 1,31) quando comparados àqueles com baixo consumo. Padrão semelhante foi observado para o consumo de refrigerantes (RP 1,28; IC95% 1,23; 1,32) e refeições do tipo fast-food (RP 1,22; IC95% 1,15; 1,29), o que evidenciou gradiente dose-resposta entre a maior frequência de consumo desses alimentos e a ocorrência de dor dentária (Tabela 3).

**Tabela 3:** Razões de prevalência (RP) e intervalos de confiança de 95% (IC95%) brutas e ajustadas da dor dentária autorreferida pelas variáveis do estudo a partir de dados da Pesquisa Nacional de Saúde do Escolar. Brasil, 2019 (n=124.898)

Consumo de alimentos ultraprocessados	Dor dentária		
	Modelo 1^a^ RP (IC95%)	Modelo 2^b^ RP (IC95%)	Modelo 3^c^ RP (IC95%)
**Consumo de alimentos ultraprocessados nas últimas 24 horas**			
Não	Referência	Referência	Referência
Sim	1,17 (1,05; 1,31)	1,26 (1,10; 1,44)	1,27 (1,10; 1,46)
**Consumo de doces nos últimos sete dias**			
Baixo (0-2 dias)	Referência	Referência	Referência
Moderado (3-4 dias)	1,09 (1,06; 1,13)	1,10 (1,06; 1,14)	1,09 (1,05; 1,13)
Alto (5-7 dias)	1,32 (1,28; 1,36)	1,28 (1,23; 1,32)	1,27 (1,22; 1,31)
**Consumo de refrigerantes nos últimos sete dias**			
Baixo (0-2 dias)	Referência	Referência	Referência
Moderado (3-4 dias)	1,16 (1,12; 1,19)	1,17 (1,13; 1,21)	1,16 (1,12; 1,21)
Alto (5-7 dias)	1,29 (1,24; 1,33)	1,28 (1,23; 1,33)	1,28 (1,23; 1,32)
**Consumo de refeições do tipo fast-food nos últimos sete dias**			
Baixo (0-2 dias)	Referência	Referência	Referência
Moderado (3-4 dias)	1,12 (1,08; 1,17)	1,12 (1,08; 1,17)	1,12 (1,08; 1,17)
Alto (5-7 dias)	1,23 (1,17; 1,29)	1,22 (1,15; 1,29)	1,22 (1,15; 1,29)

^a^não ajustado; ^b^ajustado por sexo, idade, raça/cor da pele autodeclarada, escolaridade materna; ^c^modelo 2 ajustado por visita ao dentista.

Na análise estratificada por regiões brasileiras, observou-se aumento da prevalência de dor dentária conforme a frequência de consumo de doces, refrigerantes e refeições do tipo fast-food em todas as regiões do país foi maior. Entre os adolescentes com baixo consumo de doces, a prevalência de dor variou de 16,7% na região Sul a 21,2% na região Norte. Já na categoria de alto consumo, os percentuais oscilaram de 23,0% no Sul a 27,2% no Norte. Padrão semelhante foi observado para o consumo de refrigerantes, com menores prevalências de dor entre adolescentes com baixo consumo na região Sul (17,6%) e maiores prevalências entre aqueles com alto consumo na região Norte (27,5%). Quanto ao consumo de refeições do tipo fast-food, a prevalência de dor dentária aumentou progressivamente em todas as regiões conforme a frequência de consumo foi maior. Entre os adolescentes com alto consumo, as maiores prevalências foram observadas no Sudeste (26,7%) e no Nordeste (25,6%), enquanto no Sul houve prevalência de 24,8% (Tabela 4).

**Tabela 4:** Prevalência (%) e intervalos de confiança de 95% (IC95%) de dor dentária autorreferida em adolescentes segundo frequência de consumo de alimentos ultraprocessados, doces, refrigerantes e refeições do tipo fast-food por região brasileira a partir de dados da Pesquisa Nacional de Saúde do Escolar. Brasil, 2019 (n=124.898)

Consumo de alimentos ultraprocessados	Dor dentária por regiões brasileiras				
	Norte % (IC95%)	Nordeste % (IC95%)	Sudeste % (IC95%)	Sul % (IC95%)	Centro-Oeste % (IC95%)
**Consumo de doces nos últimos sete dias**					
Baixo (0-2 dias)	21,2 (20,3; 22,0)	18,8 (18,1; 19,4)	17,3 (16,3; 18,4)	16,7 (15,6; 17,8)	18,9 (17,7; 20,2)
Moderado (3-4 dias)	23,0 (21,9; 24,1)	20,7 (19,8; 21,6)	19,3 (18,2; 20,5)	19,3 (18,0; 20,6)	20,2 (18,8; 21,8)
Alto (5-7 dias)	27,2 (26,1; 28,3)	24,9 (24,0; 25,7)	23,8 (22,6; 25,0)	23,0 (21,7; 24,4)	25,0 (23,6; 26,5)
**Consumo de refrigerantes nos últimos sete dias**					
Baixo (0-2 dias)	22,0 (21,3; 22,7)	19,8 (19,2; 20,3)	18,4 (17,5; 19,2)	17,6 (16,6; 18,5)	19,4 (18,4; 20,4)
Moderado (3-4 dias)	24,7 (23,4; 26,1)	22,6 (21,6; 23,6)	21,7 (20,3; 23,0)	21,6 (20,0; 23,3)	23,0 (21,5; 24,5)
Alto (5-7 dias)	27,5 (25,9; 29,0)	25,4 (24,0; 26,7)	24,2 (22,7; 25,7)	24,4 (22,6; 26,3)	24,9 (23,4; 26,5)
**Consumo de refeições do tipo fast-food nos últimos sete dias**					
Baixo (0-2 dias)	23,1 (22,4; 23,7)	20,4 (19,9; 20,9)	19,6 (18,8; 20,3)	19,1 (18,3; 19,9)	20,7 (19,9; 21,5)
Moderado (3-4 dias)	24,8 (23,1; 26,4)	23,3 (22,0; 24,7)	21,3 (19,6; 23,2)	21,4 (19,2; 23,9)	24,7 (22,6; 27,0)
Alto (5-7 dias)	25,0 (23,0; 27,3)	25,6 (23,7; 27,5)	26,7 (24,0; 29,7)	24,8 (21,1; 29,0)	24,5 (21,8; 27,4)

## Discussão

Este estudo identificou que maior frequência de consumo de alimentos ultraprocessados está associada a maior prevalência de dor dentária em adolescentes brasileiros, mesmo após ajuste para variáveis sociodemográficas e uso de serviços odontológicos. O consumo elevado de doces, refrigerantes e refeições do tipo fast-food apresentou associação consistente com o desfecho, o que evidenciou gradiente dose–resposta. Esses achados confirmam a hipótese inicial do estudo e reforçam a relevância da alimentação como determinante da saúde bucal na adolescência, fase marcada por consolidação de hábitos e comportamentos de risco.

Os resultados devem ser interpretados com a consideração de que a população estudada compreende adolescentes entre 13 e 17 anos, faixa etária caracterizada por alta exposição a alimentos ultraprocessados e maior autonomia alimentar, o que pode explicar a elevada prevalência de consumo observada. Por sua composição rica em açúcares livres, aditivos e carboidratos fermentáveis, esses produtos favorecem a formação e a maturação do biofilme cariogênico, o que contribui para o desenvolvimento e a progressão da cárie dentária, principal causa da dor nessa faixa etária [19,23,24]. Estudos prévios já apontaram que o consumo elevado de ultraprocessados aumenta o risco de cárie em crianças e em adolescentes [5,7], e os resultados deste estudo ampliaram essa evidência ao demostrar que também estão relacionados ao relato de dor, importante marcador de qualidade de vida [11,13]. A dor dentária, diferentemente da cárie enquanto condição clínica, configura-se manifestação direta do sofrimento e da limitação funcional causada pela doença bucal. Embora nem todas as lesões de cárie evoluam para dor, sua ocorrência geralmente reflete estágios mais avançados da doença, quando há comprometimento dentinário ou pulpar, o que desencadeia inflamação e sintomas dolorosos que repercutem no bem-estar físico e psicológico dos adolescentes [11-13]. A dor se mostra ser o indicador mais imediato da necessidade de tratamento e de impacto na qualidade de vida.

A análise regional evidenciou que a associação entre maior consumo de doces, refrigerantes e refeições do tipo fast-food e maior prevalência de dor dentária esteve presente em todas as macrorregiões do país, ainda que com variações na magnitude do efeito. As regiões Norte e Sudeste apresentaram maiores prevalências de dor dentária associada ao alto consumo de alimentos ultraprocessados, enquanto a região Sul, de modo geral, apresentou os menores percentuais. Esses contrastes possivelmente refletem as desigualdades regionais relacionadas ao padrão alimentar, ao acesso a alimentos in natura e minimamente processados e à organização dos serviços de saúde, o que destaca a importância de políticas públicas sensíveis às especificidades territoriais [25,26]. 

Adolescentes do sexo feminino, adolescentes mais velhos e aqueles com menor escolaridade materna apresentaram maior prevalência de dor dentária, padrão consistente e socialmente desigual com achados de outros estudos nacionais [12] e internacionais [3]. A associação observada com as visitas odontológicas pode refletir busca por atendimento em decorrência da dor e limitações na resolutividade da atenção prestada, o que reforça a relevância de ações preventivas [14]. A escolaridade materna, frequentemente utilizada como marcador indireto da condição socioeconômica, mostrou-se importante indicador de desigualdade, uma vez que evidências apontam que adolescentes de famílias com menor escolaridade materna e piores condições socioeconômicas tendem a consumir mais alimentos ultraprocessados [25], o que pode explicar parte das desigualdades observadas na prevalência de dor. Esses resultados reforçam que a dor dentária não deve ser compreendida apenas como um problema clínico, mas como expressão de iniquidades sociais.

Os achados deste estudo dialogam com recomendações internacionais, que orientaram limitar o consumo de alimentos ultraprocessados e açucarados a menos de 5% da ingestão calórica total diária [14,27]. A elevada frequência de consumo identificada, quase universal entre adolescentes, reforça a necessidade de estratégias educativas e regulatórias que incentivem escolhas alimentares mais saudáveis, com repercussões positivas na saúde bucal e na prevenção de doenças crônicas não transmissíveis [1,28]. A implementação de ambientes escolares saudáveis, conforme estabelecido pelo Decreto nº 11.821/2023, que estabelece diretrizes para promoção da alimentação saudável nas instituições de ensino, constitui passo importante para apoiar políticas de alimentação que protejam a saúde da população adolescente e promovam práticas intersetoriais de prevenção [29].

Entre as limitações do estudo, destacou-se o uso de medidas autorreferidas, tanto para dor quanto para consumo alimentar, sujeitas a viés de memória e percepção. A ausência de exame clínico odontológico impossibilitou a confirmação da presença de lesões de cárie, principal causa da dor dentária. O questionário alimentar, por sua vez, embora validado, não permite quantificar a ingestão ou avaliar a composição detalhada dos produtos consumidos. Foram adotadas, ainda assim, medidas diversas para minimizar possíveis vieses e fortalecer a validade dos achados, como o uso de instrumentos padronizados e validados pelo IBGE, o delineamento amostral complexo, que garantiu a representatividade nacional, e o ajuste das análises por potenciais variáveis de confusão, o que aumentou a robustez e a validade dos achados.

O consumo elevado de alimentos ultraprocessados mostrou-se consistentemente associado à maior prevalência de dor dentária em adolescentes brasileiros. Os resultados reforçaram a necessidade de estratégias de promoção da saúde e de políticas públicas que reduzam o consumo de alimentos ultraprocessados na adolescência, o que contribui para a prevenção da dor dentária e a melhoria da qualidade de vida. Estudos longitudinais futuros com inclusão de exames clínicos e análises multiníveis poderão aprofundar a compreensão das relações causais entre consumo de ultraprocessados, dor dentária e iniquidades em saúde. 

## Data Availability

Os dados utilizados neste artigo estão disponíveis em: https://osf.io/gxqdt/.
